# Investigation of quantitative susceptibility mapping in diagnosis of tuberous sclerosis complex and assessment of associated brain injuries at 1.5 Tesla

**Published:** 2020-03-11

**Authors:** Lei Zhang, Hongqiang Xue, Tao Chen, Hongzhe Tian, Xiaohu Wang, Xiaocheng Wei, Huawen Zhang, Hui Ma, Zhuanqin Ren

**Affiliations:** ^1^Department of Radiology, Baoji Hi-Tech People’s Hospital, Baoji 721013, Shaanxi, P.R. China; ^2^Department of Radiology, The First Affiliated Hospital of Xi’an Jiaotong University, Xi’an 710061, Shaanxi, P.R. China; ^3^Department of Radiology, Baoji Center Hospital, Baoji 721008, Shaanxi, P.R. China; ^4^GE Healthcare, Beijing, P.R. China; ^5^Department of Radiology, Nuclear Industry 215 Hospital of Shaanxi Province, Xianyang 712021, Shaanxi, P.R. China

**Keywords:** magnetic resonance imaging, quantitative susceptibility mapping, tuberous sclerosis complex

## Abstract

**Background and Aim:**

Tuberous sclerosis complex (TSC) is a rare disease with serious clinical consequences such as mental deficiency and epilepsy. The pathological changes of TSC include demyelination and subependymal calcified nodules. Quantitative susceptibility mapping (QSM) is a newly developed imaging technique which is capable of quantitatively measuring the susceptibility induced by iron deposition, calcification, and demyelination. The aim of this study was to investigate the use of QSM in detecting the subependymal nodules and assessing brain tissue injuries induced by cortical/subcortical tubers in TSC patients.

**Materials and Methods:**

Twelve clinically confirmed TSC patients and fifteen gender- and age-matched healthy subjects underwent measurement with conventional magnetic resonance imaging (MRI) sequences, diffusion tensor imaging (DTI), and QSM. The TSC patients further underwent a computed tomography (CT) scan. Considering CT as the ground truth, the detection rates of subependymal nodules using conventional MRI and QSM were compared by the paired Chi-square test, and the sensitivity and specificity were computed. The Bland-Altman test and independent *t*-test were performed to compare the susceptibility of cortical/subcortical regions from QSM and fractional anisotropy (FA) values from DTI between the patient and control groups, Pearson correlation was performed to examine the correlation between the susceptibility and FA values.

**Results:**

QSM was better in detecting subependymal calcified nodules compared to conventional MR sequences (*X*^2^=40.18, *P*<0.001), QSM achieved a significantly higher sensitivity of 98.3% and a lower specificity of 50%, which was compared with conventional MR sequences (46.7% and 75%, respectively). The susceptibility value of cortical/subcortical tubers in TSC patients was significantly higher than those in the control group (*t*=9.855, *P*<0.001), while FA value was lower (*t*=−8.687, *P*<0.001). Pearson correlation test revealed a negative correlation between susceptibility and FA values in all participants (*r*=−0.65, *P*<0.001).

**Conclusions:**

QSM had a similar ability in TSC compared to CT and DTI. QSM may provide valuable complementary information to conventional MRI imaging and may simplicity imaging of patients with TSC.

**Relevance for Patients:**

This study shows the feasibility of QSM to detect subependymal calcified nodules. It may provide quantitative information of white matter damage of tuberous sclerosis patients.

## 1. Introduction

Tuberous sclerosis complex (TSC) is a severe neurocutaneous syndrome that results from heterozygous mutations in either TSC1 or TSC2. TSC1 and TSC2 are located on chromosome 9q34 and 16p13, respectively [1]. It can occur in patients with of any age, with an incidence between 1/6000 and 1/10000 [2-4]. TSC can affect multiple organs, including brain, skin, kidney, heart, and lung with benign tumors. The occupant effects of benign lesions can cause secondary obstruction and organ dysfunctions by replacing essential tissue components with neoplastic tissues [5,6]. Although the morbidity has been reduced due to recent advances in treatment methods, the prognosis remains relatively poor and nearly 40% of patients died by the age of 35 [7]. At present, early diagnosis of TSC is required and it still relies primarily on the clinical manifestations. However, the clinical manifestations of different organs and systems can vary, even among closely related individuals, and the manifestations of affected organs can continue to develop over the lifespan of an individual [6], making the early diagnosis of TSC even more. Imaging plays an important role in the early diagnosis of TSC.

Brain is the most vulnerable organ in TSC. The typical TSC-related brain abnormalities include cortical/subcortical tubers, subependymal nodules, subependymal giant cell astrocytomas, and white matter lesions (radial bands) [8]. Among them, the subependymal nodules and cortical/subcortical tubers are the most two common types, with an occurrence rate of 80% and 90%, respectively [6,7]. The presence of subependymal nodules in imaging is critical evidence for accurate diagnosis for TSC [9]. The subependymal nodules in TSC patients are intraventricular protrusions comprised of abnormal cells and are typically found in the lateral ventricles adjacent to the caudate nucleus, with a high chance of containing calcification [10]. Computed tomography (CT) is commonly used for detecting subependymal nodules and is accepted as the gold standard for calcification detection [11]. Although the area of the cerebral structural abnormalities induced by cortical/subcortical tubers is stable, they are closely related to the nervous system symptoms, including epilepsy, cognitive impairment, and neurobehavioral abnormalities [7,12]. Accurate localization of cortical/subcortical tubers and assessment of the associated brain injuries has great clinical significance in treatment planning and determining the extents of excision of the epilepsy lesions, which directly impact the treatment effects, mental recovery, and patients’ life quality [13]. Previous work showed that magnetic resonance imaging (MRI) fluid-attenuated inversion recovery (FLAIR) sequence, which uses an inversion recovery pulse to suppress the cerebral spinal fluid, can be used for detecting juxtacortical and periventricular lesions [14]. FLAIR has superior ability to reveal the amount, location, and spatial extent of the cortical tubers, compared to other MR sequences [14]. Diffusion tensor imaging (DTI) can assess water molecule diffusion rates at different directions, which reflects tissue microstructures [15]. Previous work has reported significantly increased apparent diffusion rate, axial diffusivity, radial diffusivity values, and decreased fractional anisotropy (FA) values in the cortical tubers in TSC [16]. Hence, the diffusion metrics derived in DTI can provide essential information in changes of brain tissue microstructure, including hypomyelination, gliosis, and heterotopic cells induced by TSC lesions [16-18].

In addition, quantitative susceptibility mapping (QSM) was coined by de Rochefort, which can provide a quantitative spatial mapping of tissue susceptibility in the brain [19,20]. Tissue susceptibility is a macroscopic physical property which is related to its chemical composition, molecular constitution as well as the molecular and cellular structure within the tissue. Compositions such as water, myelin, iron, and calcium can affect the susceptibility of brain tissues [21]. Based on the susceptibility values, these compositions can be identified in QSM [22]. The use of multi-echo acquisition and advanced post-processing methods can improve the image signal-to-noise ratio, contrast, as well as eliminate phase aliasing caused by air, skull, and calcification [23]. This can help to better demonstrate cortical/subcortical structures and subependymal calcified nodules. At present, QSM can be used to differentiate calcification, hemorrhage, and also to analyze iron deposition and myelination quantitatively [22]. These components are closely related to the pathological compositions of TSC. To the authors’ best knowledge, the application of QSM in TSC patients has not been reported previously. Therefore, this study is designed to use QSM to (a) detect subependymal nodules and (b) assess brain tissue injuries induced by cortical/subcortical tubers in TSC patients. The goal of this study is to explore the feasibility of QSM in early diagnosis and assessment for TSC patients with atypical clinical manifestations.

## 2. Materials and Methods

### 2.1. Study participants

Clinical information of twelve patients (five males, seven females, median age 19 years, age range 12-38 years) with TSC was collected retrospectively from August 2009 to September 2016. Nine patients had seizures, four had autistic spectrum disorder, six had developmental delay, two had attention-deficit/hyperactivity disorder, and one showed no abnormal clinical manifestations. The diagnosis of TSC was performed by two experienced neurologists (over 10 years of experience). All of the patients met the established revised diagnostic criteria of TSC published by the International TSC Consensus Group in 2012 [8]. Subjects were excluded if there had incomplete clinical information or poor image quality. Sex- and age-matched control group was also recruited for comparison. The control group consisted of fifteen healthy volunteers (seven males, eight females, median age 21.5 years, age range 12-37 years). The control group showed normal in MRI and no developmental abnormality, neuropsychiatric disorder, or motor deficit. This study was approved by the institutional ethics committee and written informed consent was obtained from all the subjects.

### 2.2. Imaging protocol

All subjects underwent MRI including routine sequences (T1-weighted image [T1WI], T2-weighted image [T2WI], and T2 FLAIR), QSM and DTI on a 1.5T whole-body MR system (Signa HDe, General Electric Co., Waukesha, Wisconsin, USA) equipped with an 8-channel head coil that the patients also received a CT scan. Patients were instructed to keep still during the MRI scan. Scan parameters of the routine sequences were as following: T1WI FLAIR, repetition time (TR)/inversion time (TI)/echo time (TE)=2250/760/24 ms; T2WI, TR/TE=4200/102 ms; T2 FLAIR, TR/TI/TE=8500/1800/140 ms. All the above sequences used a field of view (FOV)=256´256 mm^2^, matrix=256´256, and slice thickness/gap=4/0.4 mm. A multi-echo gradient echo sequence was used to obtain QSM data with following parameters: Number of echoes=16; TR/TE=85/2.8-49.1 ms; echo spacing=3.1 ms; flip angle=20°; slice/gap=3/0 mm; number of average=1; FOV=256×256 mm^2^; matrix=256×256; and total scan time=302 s. DTI was acquired using a single-shot diffusion-weighted echo-planar imaging with 15 spatially isotropically arranged non-collinear directions; the scan parameters were as following: TR/TE=6175/85 ms; slice/gap=3/0 mm; FOV=256×256 mm^2^; matrix=256×256; and b-value=1000 s/mm^2^. An FA map was derived using vendor-supplied software (advantage workstation 4.5, GE Healthcare, Milwaukee, Wisconsin, USA). All the CT scans of TSC patients were performed using a 16-slice CT device (Bright Speed, General Electric Co., Milwaukee, Wisconsin, USA). The CT acquisition parameters were as following: X-ray tube current=350 mA; kVp=120 kV; slice thickness=2.5 mm; and FOV=250×250 mm^2^.

### 2.3. Data analysis

QSM was calculated using the improved orthogonal and right triangular decomposition (iLSQR) method [24]. First, CT was considered as the ground truth for the diagnosis of subependymal nodules, and one experienced radiologist assessed the amount, densities, signal intensities of non-calcified and calcified subependymal nodules with CT, conventional MRI and QSM images, separately. Second, T2 FLAIR image was considered as the ground truth for the cortical/subcortical nodules which were identified and localized on hyperintense region compared to normal-appearing white matter. To achieve consistency of the size and location of the region of interests (ROI), ROIs were first defined on T2 FLAIR by two observers independently using Mango software and then transposed to QSM and FA images for quantitative susceptibility and FA measurements. For normal controls, the susceptibility and FA values were measured at the matched region with the patient group by the same two observers independently. If TSC patients showed multiple lesions, a maximal of five measurements were obtained on a single patient.

### 2.4. Statistical analysis

Statistical analysis was performed using SPSS 17.0 (SPSS, Chicago, IL, USA). To consider CT as the ground truth, the detection rates of subependymal nodules using conventional MR sequences and QSM were compared by the paired Chi-square test, and the sensitivity and specificity were computed. The inter-observer consistency of the susceptibility values of cortical/subcortical regions in TSC patients and control subjects was assessed by Bland-Altman analysis. Independent *t*-test was performed to compare the susceptibility and FA values between the patient and control groups; Pearson correlation was performed to examine the correlation between the susceptibility and FA values of cortical/subcortical regions in TSC patients and control subjects. *P*<0.05 was considered as statistically significant.

## 3. Results

### 3.1. Subependymal nodules

**Figure 1:**
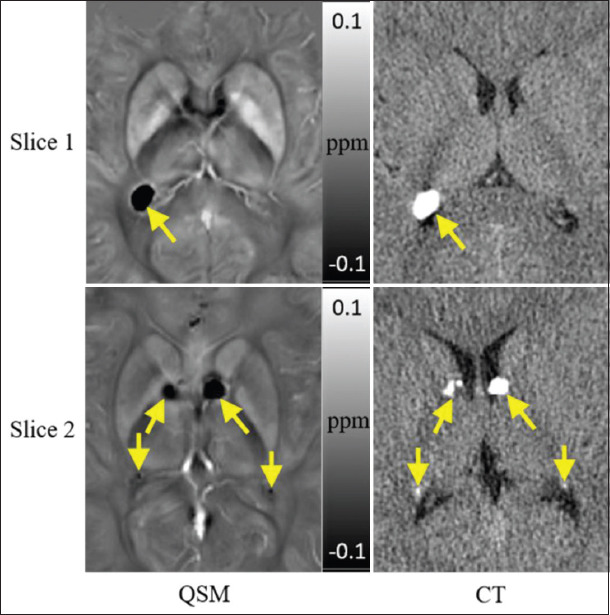
Quantitative susceptibility mapping and computed tomography showing calcified subependymal nodules from a 27-year-old male patient. Both imaging modalities could demonstrate all of the calcification.

A total of 68 subependymal nodules were identified in twelve TSC patients, among which eight lesions were non-calcified and 60 lesions were calcified (Table 1). QSM was better in detecting calcified subependymal nodules compared to conventional MR sequences (*X*^2^=0.18, *P*<0.001). The conventional MR sequences achieved a lower sensitivity of 46.7% and higher specificity of 75%. Conventional MR sequences were beneficial in revealing the non-calcified and mature calcified subependymal nodules, as non-calcified subependymal nodules are seen as iso-intense compared to normal-appearing white matter in T1WI, T2WI, and T2 FLAIR, whereas mature calcified subependymal nodules are seen isointense or slightly hyperintense in T1WI, slightly hypointense on T2WI, and iso-intense on T2 FLAIR. Conventional MR sequences were not able to demonstrate micro and immature calcified subependymal nodules clearly due to inadequate image contrast, while QSM achieved a significantly higher sensitivity of 98.3% and lower specificity of 50%. QSM could clearly reveal almost all of the calcified subependymal nodules which showed hypointense. Figure 1 shows examples of calcified subependymal nodules in both QSM and CT. Pearson Chi-square test showed similar performance of QSM and CT in revealing calcified subependymal nodules (*X*^2^=1.01, *P*=0.315).

**Table 1:** Detection rates of subependymal nodules in TSC.

Subependymal nodules	CT	Conventional. MRI	QSM
Calcified			
True positive calcified	60	28	59
False positive calcified	0	2	4
Non-calcified			
True negative non-calcified	8	6	4
False negative non-calcified	0	32	1

*CT: Computed tomography; Convent. MRI: Conventional magnetic resonance imaging, QSM: Quantitative susceptibility mapping, TSC: Tuberous sclerosis complex

### 3.2. Cortical/subcortical nodules

**Figure 2:**
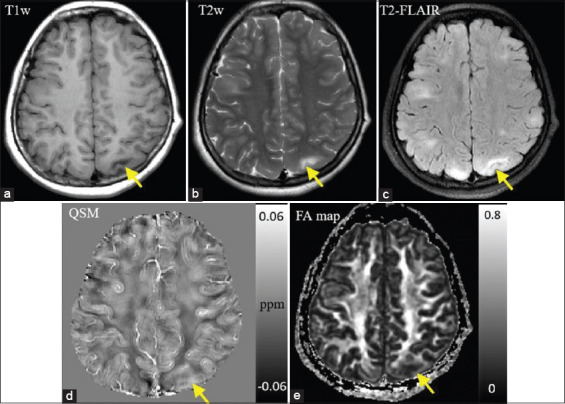
(a-e) Example of cortical/subcortical nodules from a 16-year-old female patient. The tuberous sclerosis complex lesions appeared hypo- or iso-intense on T1-weighted image, hyperintense on T2-weighted image, and T2-fluid-attenuated inversion recovery. The lesions had hyperintense on quantitative susceptibility mapping and hypointense on fractional anisotropy map.

**Figure 3:**
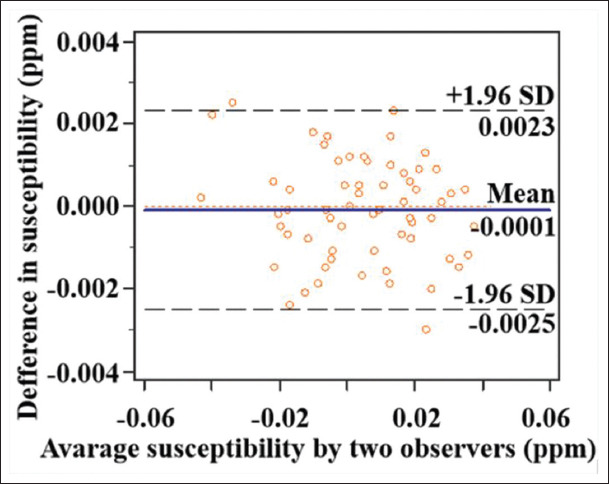
Bland-Altman test of the susceptibility values of cortical/subcortical regions in tuberous sclerosis complex patients and control subjects measured by two observers. The figure showed that the differences between measurements from the two observers were very close to 0 ppm, only one scatter was located outside the limit of agreement, which represents a portion <5%.

**Figure 4:**
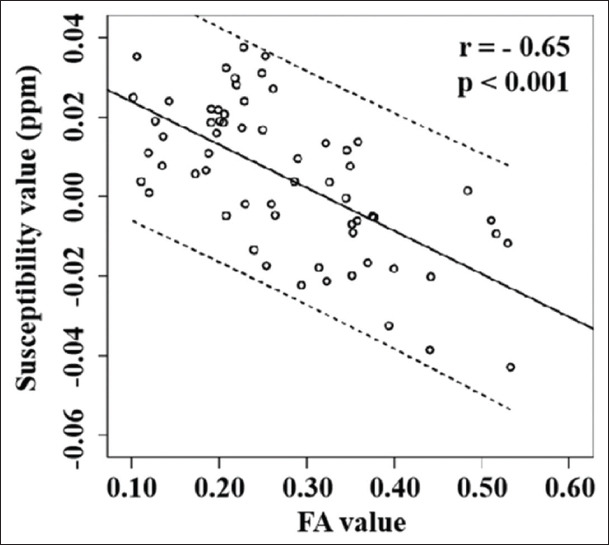
Scatter plot of the fractional anisotropy (x-axis) and susceptibility (y-axis) values of the cortical/subcortical regions in all the subjects. Solid lines represent the fitting curve and dotted lines represent the 95% confidence interval.

Cortical/subcortical nodules showed hypo- or iso-intense compared to normal appearing gray matter in T1WI, slightly hyperintense in T2WI, and T2 FLAIR (Figure 2a-c). T2 FLAIR could better reveal the cortical/subcortical nodules in the brain than T1WI and T2WI. The nodules showed a hyperintense signal in QSM (Figure 2d), which had a similar appearance in T2 FLAIR. The nodules also showed a hypointense signal in FA maps [Figure 2e]. Susceptibility and FA values were measured in 31 cortical/subcortical lesions from twelve patients. Similar measurements were also obtained in 31 regions at matched locations from fifteen control subjects. Bland-Altman test showed good consistency between the susceptibility measurements from two observers (Figure 3), the limit of agreement (± 1.96×SD) was – 0.003 ppm and 0.002 ppm, and the average difference (mean) was close to 0 ppm. TSC patients had significantly higher susceptibility and lower FA values in cortical/subcortical tubers compared to control subjects (Table 2). There was a negative correlation between the susceptibility and FA values in all the subjects (*r*=−0.65, *P*<0.001) (Figure 4). The results proved that susceptibility and FA values showed similar ability in assessing TSC induced brain injuries.

**Table 2:** Susceptibility and FA value of cortical/subcortical nodules in patients and control group (mean ± SD).

Cortical/subcortical nodules	Susceptibility value (ppm)	FA value
Patients group (*n*=31)	0.019±0.010	0.194±0.058
Control group (*n*=31)	−0.010±0.013	0.360±0.089
*t*-value	9.855	−8.687
*P*-value	<0.001	<0.001

FA: Fractional anisotropy

## 4. Discussion

The subependymal nodules and cortical/subcortical tubers are the most two commonly manifestations in nervous system, they are important in clinical diagnosis of TSC [6,8,14]. Imaging of these manifestations is the basis of early diagnosis and assessment of the TSC induced brain injuries, which are important for treatment planning [11]. The subependymal nodules of the ventricle often locate on the lateral ventricle wall adjacent to the caudate nucleus, with a high calcification rate up to 88% [7,25]. CT is commonly used as the gold standard for examining calcification. Calcified nodules are identified with a CT value higher than 100 Hu [26]. In QSM, calcification shows hypointense as it is diamagnetic [21,27,28]. QSM utilizes regularization, data weighting, and background field removal to remove phase ambiguity in the image and therefore, can directly demonstrate tissue susceptibility [20,29]. In this study, large calcified subependymal nodules had clear boundaries in the QSM images. The amount, location, size, and morphology of calcified nodules adjacent to the lateral ventricle were clearly shown in QSM, which is in agreement with the previous finding [30]. QSM had similar performance in identifying calcified subependymal nodules with CT, but the specificity of QSM is relatively low. The reason is possible that the non-calcified subependymal nodules adjacent to the medullary vein or choroid plexus, thereby reducing the ability of QSM to distinguish calcification. With the help of other conventional MR sequences in identifying non-calcified nodules, the combination of QSM and conventional MR sequences can significantly increase the detection rate of subependymal nodules compared to CT. The combination of QSM and conventional MR sequences is preferable as a radiation-free screening and follow-up imaging method for TSC patients, especially in young adults and children [31], this method has high sensitivity.

The cortical/subcortical lesions contain a various concentration of abnormal giant cells, which have both glial and neuronal characteristics, can induce gliosis, hypomyelination, neurons arrangement disorder, and destroy local tissue microstructure [31]. These lead to a series of severe central nervous system manifestations, including epilepsy, cognitive dysfunction, and autism [32]. Accurate imaging and quantitative analysis of cortical/subcortical nodules are important for early diagnosis and treatment planning of TSC. Conventionally, diffusion metrics derived from DTI are used to assess TSC-related brain injuries. It has been shown that FA value can be used to assess cortex and subcortex microstructure change, which is closely related to demyelination [16]. As myelin, the lipoprotein sheath surrounding axons, is more diamagnetic than water molecules in cerebrospinal fluid, demyelination would cause local susceptibility variations [21]. This study showed that in cortical/subcortical nodules, (a) TSC patient has higher susceptibility value compared to control group and (b) the susceptibility value is negative correlated to FA value in all participants. The increased susceptibility in the local cerebral region may due to the interaction of hyperplasia of glial cells and white matter demyelination in the cortex and subcortex, which changes the local microstructure in the brain. Therefore, the susceptibility value derived from QSM could be a reliable biomarker for assessing the brain tissue injuries induced in cortical/subcortical lesions.

There are several limitations in this study. First, this study only measured the susceptibility of the cortical/subcortical lesions, the susceptibility of normal-appearing white matter and deep nuclei grey matter can be investigated in the future study which may help better evaluation of the diseases. Second, the relationship between the susceptibility value of cortical/subcortical nodules and cognitive function or autism for TSC patients can be further explored to provide evidence for the confirmation of epilepsy lesions. Third, the number of patients enrolled in this study was constrained due to the rareness of TSC.

## 5. Conclusion

To the best of our knowledge, this is the first study to apply QSM to detect subependymal nodules and assess brain injuries induced by cortical/subcortical tubers in TSC patients. The excellent inter-rater agreements shown in this study demonstrate the susceptibility measurements have good reproducibility and may be easily adopted by clinical use. In conclusion, TSC often progresses to serious clinical consequences which had a close relationship with cortical/subcortical nodules and white matter lesions. QSM is a newly developed imaging technique which is capable of quantitatively measure the susceptibility induced by iron deposition, calcification, and demyelination. Our results suggest that QSM had similar ability in the subependymal calcified nodules compared to CT, and the quantitative evaluation of white matter damage compared to DTI. Hence, QSM may have a valuable complementary role into conventional MRI imaging and may simplicity imaging of patients with TSC.
